# Simple, accurate calculation of mechanical power in pressure controlled ventilation (PCV)

**DOI:** 10.1186/s40635-022-00448-5

**Published:** 2022-05-30

**Authors:** Christine A. Trinkle, Richard N. Broaddus, Jamie L. Sturgill, Christopher M. Waters, Peter E. Morris

**Affiliations:** 1 Department of Mechanical Engineering, College of Engineering, University of Kentucky, 277 Ralph G. Anderson Building, Lexington, KY 40506 USA; 2 Division of Pulmonary, Critical Care and Sleep Medicine, College of Medicine, University of Kentucky, Lexington, KY USA; 3 Department of Physiology, College of Medicine, University of Kentucky, Lexington, KY USA; 4 Saha Cardiovascular Research Center, University of Kentucky, Lexington, KY USA

**Keywords:** Mechanical ventilation, Mechanical power equation, Pressure-controlled ventilation

## Abstract

**Background:**

Mechanical power is a promising new metric to assess energy transfer from a mechanical ventilator to a patient, which combines the contributions of multiple parameters into a single comprehensive value. However, at present, most ventilators are not capable of calculating mechanical power automatically, so there is a need for a simple equation that can be used to estimate this parameter at the bedside. For volume-controlled ventilation (VCV), excellent equations exist for calculating power from basic ventilator parameters, but for pressure-controlled ventilation (PCV), an accurate, easy-to-use equation has been elusive.

**Results:**

Here, we present a new power equation and evaluate its accuracy compared to the three published PCV power equations. When applied to a sample of 50 patients on PCV with a non-zero rise time, we found that our equation estimated power within an average of 8.4% ± 5.9% (mean ± standard deviation) of the value obtained by numerical integration of the *P*–*V* loop. The other three equations estimated power with an error of 19.4% ± 12.9% (simplified Becher equation), 10.0% ± 6.8% (comprehensive Becher equation), and 16.5% ± 14.6% (van der Meijden equation).

**Conclusions:**

Our equation calculates power more accurately than the other three published equations, and is much easier to use than the only previously published equation with similar accuracy. The proposed new mechanical power equation is accurate and simple to use, making it an attractive option to estimate power in PCV cases at the bedside.

## Background

Mechanical power is a promising new metric to evaluate ventilator settings using a single comprehensive value that captures the influence of multiple static and dynamic metrics—positive end-expiratory pressure (PEEP), lung compliance, respiratory rate, and others—resulting in an encompassing picture of energy transfer from a ventilator to the patient [1]. However, many ventilators are not capable of calculating power*.* While equations for volume-controlled ventilation (VCV) exist for calculating mechanical power from basic ventilator parameters [2, 3], there remains an opportunity for developing simplified equations for pressure-controlled ventilation (PCV) that can be used at the bedside.

Becher et al*.* [4] presented two solutions to the PCV power estimation problem. Their simplified equation modeled pressure during inspiration as a square wave—removing rise time. As the authors noted, however, this leads to decreased accuracy when rise time is not equal to zero. Their comprehensive equation accounts for a non-zero rise time, increasing accuracy, but like all complex equations may be challenging for bedside application. Recently, van der Meijden et al*.* [5] presented a simplified equation, but we and others [6] found that it produces lower accuracy than the comprehensive Becher equation. To address this limitation, we developed a new simplified equation that can be applied at the bedside, and we evaluated its accuracy using data from patients on PCV.

## Results

We developed a new simplified equation for patients on PCV, where pressure increases from the end-expiratory value (${P}_{\mathrm{PEEP}}$, [cmH_2_O]) to a maximum pressure (${P}_{\mathrm{PEEP}}+{\Delta P}_{\mathrm{insp}}$, [cmH_2_O]) over a prescribed rise time (${t}_{\mathrm{slope}}$, [s]). The result can be represented using a linear model over the entire realistic range of ventilator settings and patient parameters, resulting in a simple, low-error equation for mechanical power (${\mathrm{MP}}_{\mathrm{LM}}$, [J/min]):

1 $${\mathrm{MP}}_{\mathrm{LM}}=0.098\cdot \mathrm{RR}\cdot \left\{{V}_{\mathrm{T}}\cdot \left({P}_{\mathrm{PEEP}}+{\Delta P}_{\mathrm{insp}}\right)-0.15\cdot {{\Delta P}_{\mathrm{insp}}}^{2}\cdot {t}_{\mathrm{slope}}/R\right\}$$

where $\mathrm{RR}$ is respiratory rate (breaths/min), ${V}_{\mathrm{T}}$ is tidal volume (L), ${\Delta P}_{\mathrm{insp}}$ is pressure change from end expiration to end inspiration (cmH_2_O), and $R$ is flow resistance [cmH_2_O/(L/s)]. The complete derivation is included in “Methods” section.

To evaluate the accuracy of our equation and the three published power equations, we used mechanical ventilator data from 50 critically ill patients on PCV who had been admitted to an academic medical center. Details of patient and ventilator parameters for the patients can be found in “Methods” section (Tables 1, 2). For each patient, we integrated the *P*–*V* curve numerically to obtain the integrated mechanical power value (${\mathrm{MP}}_{\mathrm{ref}}$):

**Table 1:** Selected patient parameters (*N* = 50 subjects)

Patient characteristics	Mean value (interquartile range)
Age (years)	59.4 (24.0–84.0)
Sex male	28 (56%)
BMI (kg/m^2^)	32.8 (16.5–66)
IBW (kg)	63.3 (43.1–80.0)
Total vent days	16.4 (2.0–61.0)
P/F ratio	210.6 (81.0–680.0)
COPD	7 (14%)
Diabetes	19 (38%)
Cancer	8 (16%)
COVID-19	17 (34%)
ARDS	27 (59%)
Pneumonia	26 (52%)
Shock	27 (54%)
Paralytic use	1 (2%)
Patient spontaneously breathing	21 (42%)

**Table 2:** Selected ventilator parameters (*N* = 50 subjects)

Ventilator characteristics	Mean value (interquartile range)
*V*_T_/IBW (mL/kg)	7.622 (5.2–10.9)
*V*_min_ (L/min)	10.4 (4.8–17.4)
Respiratory rate	22 (14.0–36.0)
Driving pressure	14.4 (5.0–29.0)
PEEP (cmH_2_O)	7.7 (5.0–18.0)
*C*_dyn_ (mL/cmH_2_O)	38.7 (16.0–137.0)
Inspiratory time	0.9 (0.6–1.2)

2 $${\mathrm{MP}}_{\mathrm{ref}}=0.098 \cdot \mathrm{RR}\cdot {\int }_{0}^{{V}_{\mathrm{T}}}{P}_{\mathrm{aw}}\mathrm{d}V$$

Mechanical power was also calculated using our Linear Model (LM, Eq. 1), the simplified Becher (sB) [4], comprehensive Becher (cB) [4], and van der Meijden (vdM) [5] equations:

3 $${\mathrm{MP}}_{\mathrm{sB}}=0.098\cdot \mathrm{RR}\cdot \left\{{V}_{\mathrm{T}}\cdot \left({P}_{\mathrm{PEEP}}+{\Delta P}_{\mathrm{insp}}\right)\right\}$$

4 $${\mathrm{MP}}_{\mathrm{cB}}=0.098\cdot \mathrm{RR}\cdot \left\{{V}_{\mathrm{T}}\cdot \left({P}_{\mathrm{PEEP}}+{\Delta P}_{\mathrm{insp}}\right)-{{\Delta P}_{\mathrm{insp}}}^{2}\cdot C \cdot \left[0.5-\frac{R\cdot C}{{t}_{\mathrm{slope}}}+{\left(\frac{R\cdot C}{{t}_{\mathrm{slope}}}\right)}^{2}\cdot \left(1-{e}^{-{t}_{\mathrm{slope}}/(R\cdot C}\right)\right]\right\}$$

5 $${\mathrm{MP}}_{\mathrm{vdM}}=0.098\cdot \mathrm{RR}\cdot {V}_{\mathrm{T}}\cdot \left\{{P}_{\mathrm{PEEP}}+{\Delta P}_{\mathrm{insp}}\cdot \left(1-{e}^{-{t}_{\mathrm{insp}}/\left(R\cdot C\right)}\right)\right\}$$

Here, ${t}_{\mathrm{insp}}$ is the total inspiratory time (s) and $C$ is compliance (L/cmH_2_O). Values for patient parameters were calculated using the downloaded patient ventilator data, including $R$ and $C$ values which were calculated using the least-squares method [7].

The four power equations estimated power within the following (mean ± standard deviation) percent error of the numerically integrated value: our linear model equation (8.4% ± 5.9%), the simplified Becher equation (19.4% ± 12.9%), the comprehensive Becher equation (10.0% ± 6.8%), and the van der Meijden equation (16.5% ± 14.6%). A paired samples *t* test analysis of the error values indicates a statistically significant difference between the error values of the linear model and sB (*p* < 0.0001), between the linear model and cB (*p* < 0.005), and between the linear model and vdM (*p* < 0.0001).

**Fig. 1:**
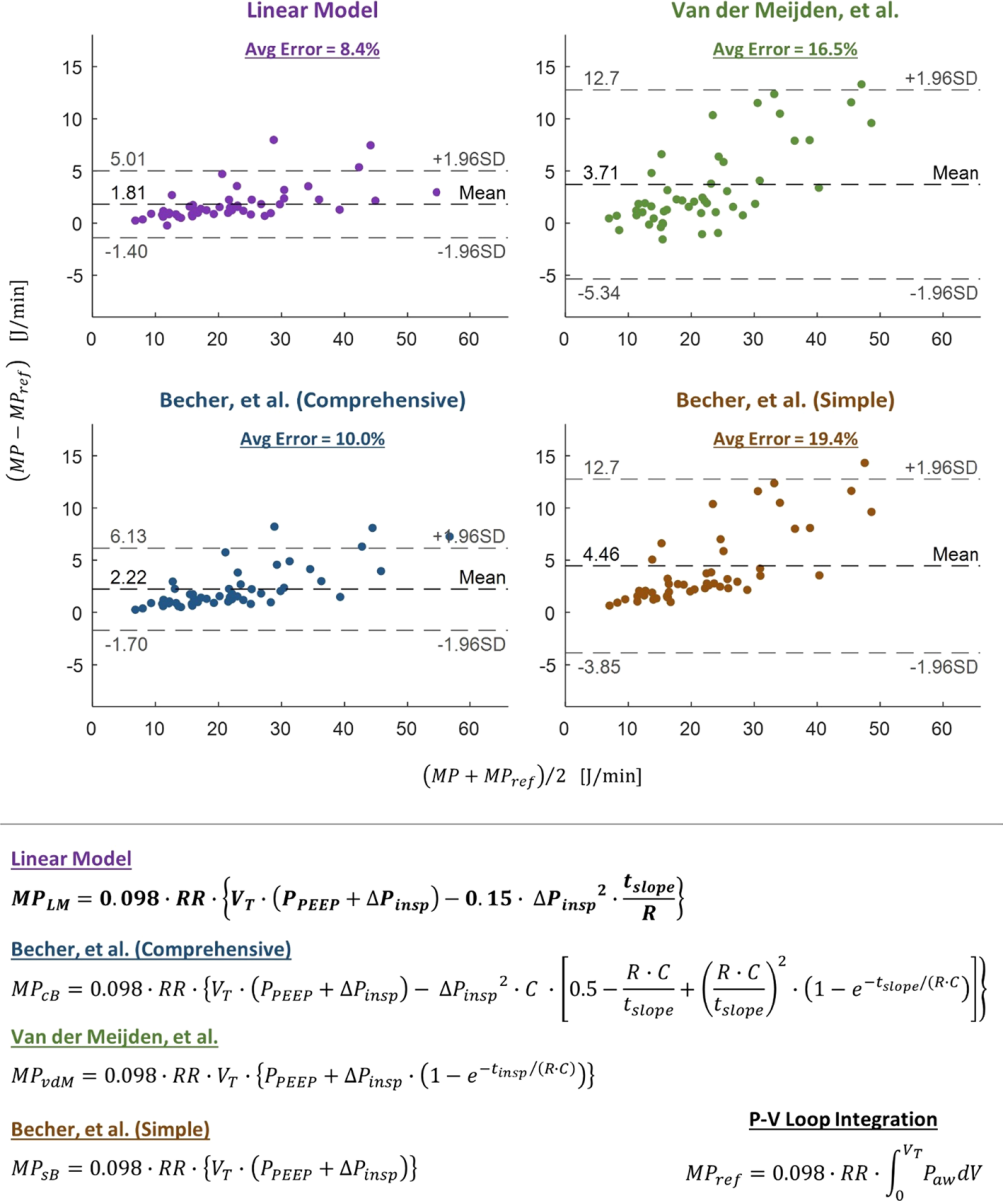
Four equations for estimating mechanical power in PCV patients (bottom), and Bland–Altman plots for each of these equations [4, 5] (top). Bland–Altman plots were generated by plotting the mean of the calculated value (MP) and the numerically integrated value (MP_ref_) for each equation (*x*-axis) against the difference between the calculated value and numerically integrated value (*y*-axis)

We performed a Bland–Altman analysis on the resulting data, comparing the results of each equation to the numerically integrated values. The limits of agreement (LoA) for our equation were − 1.4 to + 5.01 J/min with a bias of 1.81 J/min; the LoA and bias for each of the other three equations were: − 5.34 to 12.7 J/min (bias: 3.71 J/min) for vdM, − 1.70 to 6.13 J/min (bias: 2.22 J/min) for cB, and − 3.85 to 12.7 J/min (bias: 4.46 J/min) for sB. Bland–Altman plots including the LoA and bias for each equation are shown in Fig. 1. Details on the calculation of percent error and the limits of agreement can be found in the data analysis portion of “Methods” section.

**Fig. 2:**
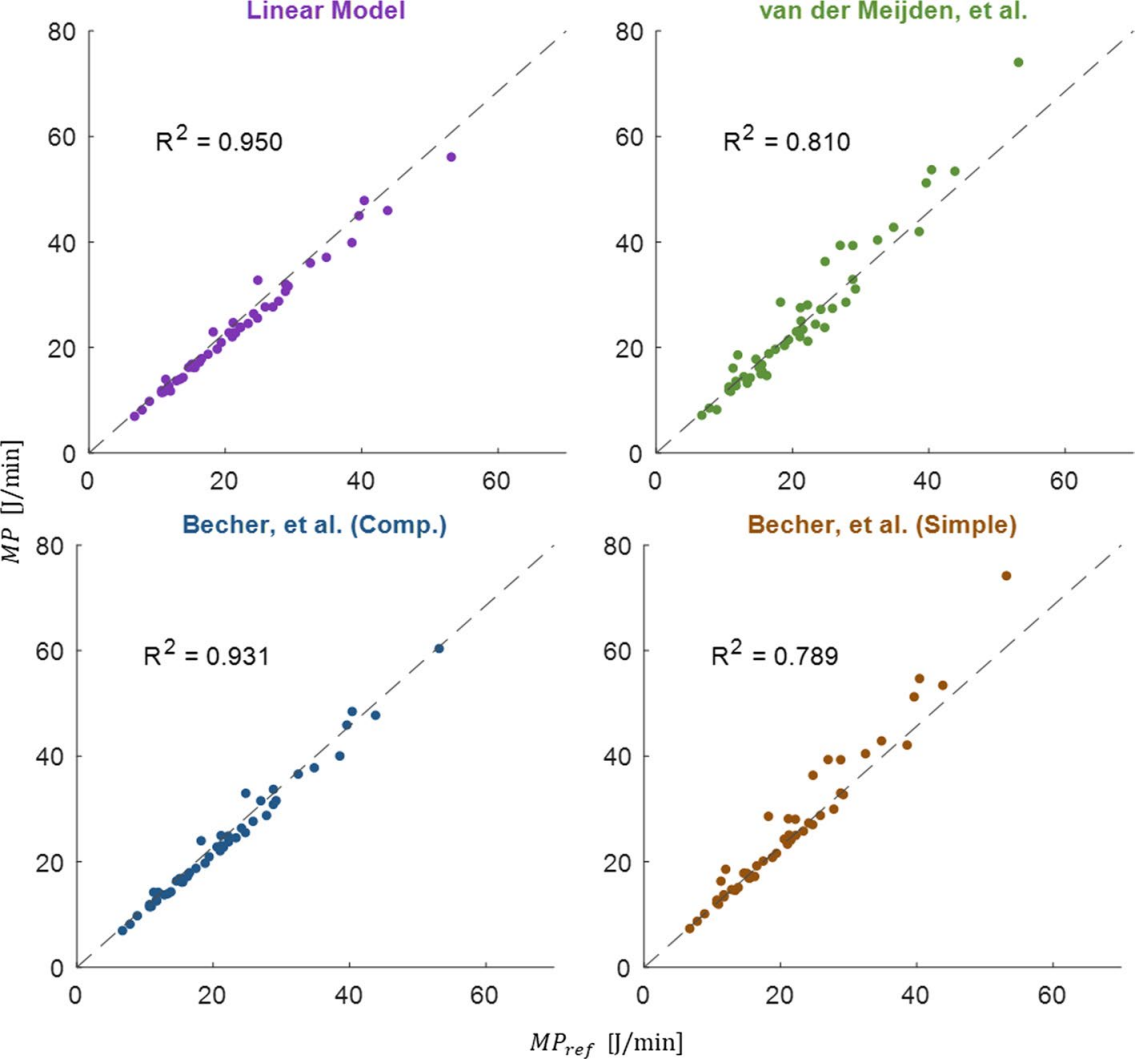
Agreement between each of the four mechanical power equations (*y*-axis) and the numerically integrated value (MP_ref_) (*x*-axis). Dashed line represents 1:1 slope

Plots showing agreement between the integrated mechanical power value and the values calculated using each equation are given in Fig. 2. Agreement with the “gold standard” integrated power values was strongest using our LM equation (coefficient of determination, *R*^2^ = 0.950), followed by the comprehensive Becher (*R*^2^ = 0.931), van der Meijden (*R*^2^ = 0.810), and simplified Becher (*R*^2^ = 0.789) equations.

**Fig. 3:**
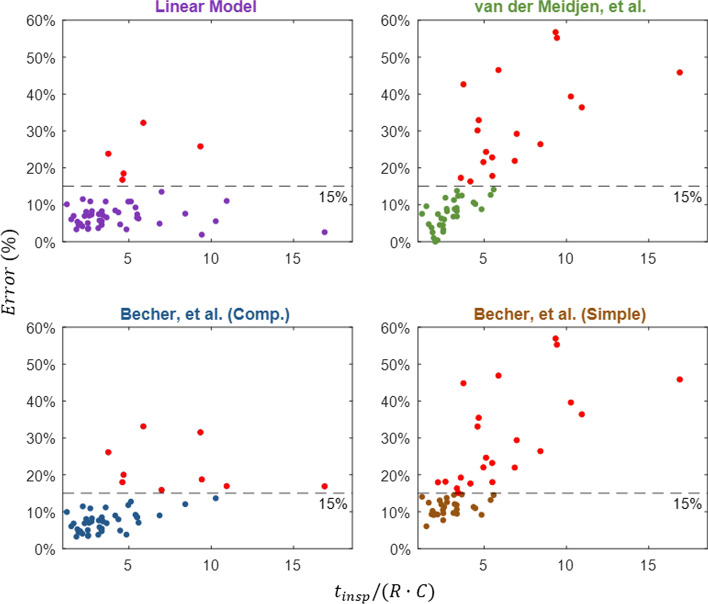
Percent error (MP_ref_ − MP)/MP_ref_ for each of the four equations [2, 3] as a function of the parameter *t*_insp_/(*R*·*C*). Calculations that resulted in greater than 15% error are highlighted in red

The errors for sB and vdM were found to be higher especially in patients, where the quantity ${t}_{\mathrm{insp}}/(R\cdot C)$ is large: i.e., patients with long inspiratory time (${t}_{\mathrm{insp}}$), low flow resistance ($R$), and/or low compliance ($C$), as shown in Fig. 3. Patients in our data set, where this parameter was above 5.5 (the top 20% of the patient values), the average percent error for vdM and sB was 37.2% and 37.3%, compared with 17.5% for cB and 11.1% for LM.

## Discussion

Numerical integration of the *P-V* loop (${\mathrm{MP}}_{\mathrm{ref}}$, Eq. 2) represents the “gold standard” for accurate calculation of mechanical power transferred from a ventilator to a patient. However, many ventilators—particularly older models—lack the ability to calculate power directly. Without this built-in capability, numerical integration is a cumbersome process requiring hardware and software that is not easily accessible to the vast majority of medical practitioners.

The process of off-board numerical integration generally requires the following steps: (1) downloading raw data files from ventilators (which often requires purchasing custom hardware to link to older models); (2) converting the data from the original format to vector files; (3) manually isolating individual *P-V* loops in the data; and (4) analyzing the data using a numerical integration program. In our experience, the entire process requires multiple people with unique areas of expertise and can take upwards of 15–30 min per patient data set. Therefore, while we anticipate that all ventilators will eventually calculate power automatically at the bedside, at present, there is still a need for accurate equations to estimate mechanical power. This clinically unmet need has been heightened recently given the exorbitant number of patients requiring mechanical ventilation due to the SARS-CoV-2 pandemic.

There is also an inherent value in having accurate, predictive equations for calculating mechanical power instead of relying solely on numerical integration. Equations such as ours allow practitioners to estimate the effects of changing certain ventilator parameters on mechanical power, prior to physically changing these parameters at the bedside. Three of the four equations discussed here (LM, cB, vdM) require patient parameters to calculate ventilator power; in contrast, and sB can be calculated with only ventilator parameters (${V}_{\mathrm{T}}, {P}_{\mathrm{PEEP}},\Delta {P}_{\mathrm{insp}}, \mathrm{RR}$), making it easy to apply, even without knowledge of an individual patient. However, this lack of patient-specificity may be one of the reasons that sB consistently generated the highest error of the four equations.

The LM equation presented here is a function of ventilator parameters and $R$, while cB and vdM require both $R$ and $C$. Because flow resistance and compliance are patient-associated parameters, they cannot be calculated a priori, so ventilator-associated data for an individual patient is required to use any of these three equations. It should also be noted that $R$ and $C$ are likely not constants with respect to ventilator parameters, so caution should be used when assuming a constant value for $R$ and/or $C$, especially if using ventilator parameters that are substantially different from those for which the patient parameters were originally calculated.

Of the published mechanical power equations, only the simplified Becher equation (sB) is easier to apply than the equation proposed here (${\mathrm{MP}}_{\mathrm{LM}}$, Eq. 1). However, while the sB equation is quite simple, it neglects the contribution of rise time and patient parameters to mechanical power; when applied to our patient data set, the sB equation produces almost twice the average error of our proposed equation. The comprehensive Becher equation is similar in accuracy to ours, but is significantly more complex. Finally, the van der Meijden equation is similar in complexity, but, like the sB equation, produces close to twice the error on average. Therefore, we believe that the new Linear Model mechanical power equation presented here fills an important need—providing a combination of accuracy and simplicity that can be useful in bedside and research applications.

In addition, the data used to evaluate all equations include non-paralyzed patients, while others [2, 3] may have exclusively included patients who were sedated without spontaneous breathing. As demonstrated here, our equation combines the simplicity of the sB and vdM equations with the accuracy of the cB equation, providing an efficient option for bedside calculation of mechanical power that can be used whether or not paralytic medications are currently in use. However, it should be noted that in cases where patient breathing effort is nonzero, there will be mechanical power contributions from the both the ventilator and the patient's respiratory muscles. None of the equations presented here, including ours, distinguish between these contributions.

## Conclusions

Mechanical power is a promising new parameter that may help determine lung-protective strategies in ventilation. However, since most currently available adult ventilators do not calculate mechanical power, there is a need for an easy-to-use equation that can be applied at the bedside. The linear model equation we presented here has an excellent combination of simplicity and accuracy—providing a more accurate estimation of mechanical power in our PCV patient data sets than any other published equation.

## Methods

### Derivation of mechanical power equation

Mechanical power ($\mathrm{MP}$) can be calculated as the integral of the airway pressure (${P}_{\mathrm{aw}},$ [cmH_2_O]) over volume ($V$) during inspiration, or

6 $$\mathrm{MP}={\int }_{0}^{{V}_{\mathrm{T}}}{P}_{\mathrm{aw}}\mathrm{d}V={\int }_{0}^{{t}_{\mathrm{insp}}}{P}_{\mathrm{aw}}\cdot \frac{\mathrm{d}V}{\mathrm{d}t}\mathrm{d}t$$

where ${V}_{\mathrm{T}}$ represents the tidal volume and ${t}_{\mathrm{insp}}$ is the total inspiratory time. For a simple approximation of mechanical power during pressure controlled ventilation, the airway pressure can be assumed to have a constant value of ${P}_{\mathrm{aw}}={P}_{\mathrm{PEEP}}+\Delta {P}_{\mathrm{insp}}$ during inspiration, where ${P}_{\mathrm{PEEP}}$ is the positive end expiratory pressure and $\Delta {P}_{\mathrm{insp}}$ is the change in pressure from the end of expiration to the end of inspiration. This results in the “simple” power formula reported by Becher [4]. This can be a reasonable estimation, but incurs error in many cases—particularly when rise time at the beginning of inspiration is non-zero.

To account for non-zero rise time, we assume that the driving pressure waveform takes the same form as assumed by Becher [4] in the derivation of their comprehensive power equation. Briefly, this assumes that at the beginning of each inspiration the airway pressure increases linearly from ${P}_{\mathrm{aw}}={P}_{\mathrm{PEEP}}$ at time $t=0$ to ${P}_{\mathrm{aw}}={P}_{\mathrm{PEEP}}+\Delta {P}_{\mathrm{insp}}$ at time $t={t}_{\mathrm{slope}}$:

7 $${P}_{\mathrm{aw}}=\left\{\begin{array}{cc}{P}_{\mathrm{PEEP}}+\Delta {P}_{\mathrm{insp}}\cdot \frac{t}{{t}_{\mathrm{slope}}}& \mathrm{when}\, 0\le t<{t}_{\mathrm{slope}}\\ {P}_{\mathrm{PEEP}}+\Delta {P}_{\mathrm{insp}}& \mathrm{when}\, t\ge {t}_{\mathrm{slope}}\end{array}\right.$$

Using this assumption, it is possible to derive the “comprehensive” mechanical power equation presented by Becher [4] as Eq. (4).

To account for the most common units of these parameters in clinical practice, this equation includes a conversion factor of 0.098 Pa m^3^/(L cmH_2_O). It assumes volumes in liters, pressures in cmH_2_O, respiratory rate in breaths per minute, time in seconds, compliance ($C$) in L/cmH_2_O, and flow resistance ($R$) in cmH_2_O/(L/s).

While this equation is more accurate than the simple equation, it is complex, and, therefore, challenging to implement in practice—particularly at the bedside. To simplify this equation, it is possible to derive a linear approximation of Eq. (4) for all reasonable values of ventilator and patient parameters. To do so, first we define the term in the square brackets in Eq. (4) as

8 $$f\left(\frac{{t}_{\mathrm{slope}}}{R\cdot C}\right)=0.5-\frac{R\cdot C}{{t}_{\mathrm{slope}}}+{\left(\frac{R\cdot C}{{t}_{\mathrm{slope}}}\right)}^{2}\cdot \left(1-{e}^{-\frac{{t}_{\mathrm{slope}}}{R\cdot C}}\right)$$

Or, making the substitution ${x=t}_{\mathrm{slope}}/(R\cdot C)$, this becomes

9 $$f\left(x\right)=0.5-\frac{1}{x}+\frac{1}{{x}^{2}}\cdot \left(1-{e}^{-x}\right)$$

For a defined range of $x$ values, $f(x)$ can be approximated by a linear function, $g(x)$. If the range over which the linear approximation is made is ${x}_{1}\le x\le {x}_{2}$, one reasonable linear function can be defined as follows:

10 $$g\left(x\right)=\frac{f\left({x}_{2}\right)-f({x}_{1})}{{x}_{2}-{x}_{1}}\cdot \left(x-{x}_{1}\right)+f({x}_{1})$$

For pressure controlled ventilation, a reasonable lower limit is ${x}_{1}=0$, which corresponds to an inspiratory rise time of zero (${t}_{\mathrm{slope}}=0$). A reasonable upper limit is ${x}_{2}=0.5$, which can be achieved in a number of ways, including ${t}_{\mathrm{slope}}=150\, \mathrm{msec}$, $R=10\, {\mathrm{cmH}}_{2}\mathrm{O}/\mathrm{L}/\mathrm{s}$, and $C=0.03\, \mathrm{L}/{\mathrm{cmH}}_{2}\mathrm{O}$. Decreasing the value of ${t}_{\mathrm{slope}}$ or increasing the values of $R$ or $C$ would all have the same effect of decreasing $x$, thus we propose that $0\le x\le 0.5$ represents a reasonable operating range for $x$. In practice, the equation holds for a range larger than this with minimal error. Therefore, we can approximate $f\left(x\right)$ using the following:

11 $$g\left(x\right)=\frac{f\left(0.5\right)-f(0)}{0.5-0}\cdot \left(x-0\right)+f\left(0.5\right)$$

12 $$g\left(x\right)\approx 0.15\cdot x$$

**Fig. 4:**
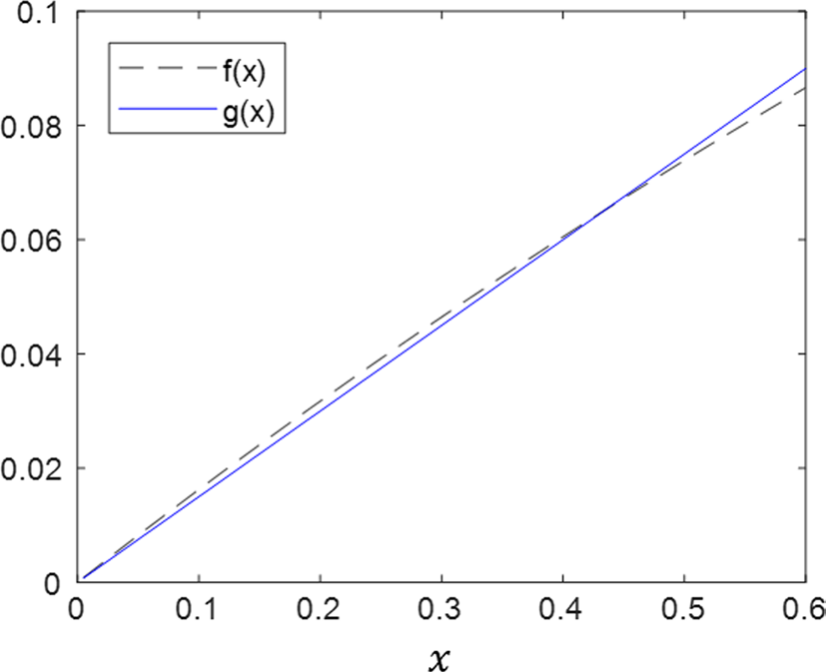
Comparison of original function, *f*(*x*), and linear approximation, *g*(*x*). Functions show excellent agreement over a realistic range of *x* values

Figure 4 plots Eqs. (9) and (12) over a range of $x$ values, showing good agreement between the original function ($f\left(x\right)$) and the linear approximation ($g\left(x\right)$).

Substituting Eq. (12) into Eq. (4) in place of $f(x)$ yields

13 $$\mathrm{MP}=0.098\cdot \mathrm{RR}\cdot \left\{{V}_{\mathrm{T}}\cdot \left({P}_{\mathrm{PEEP}}+{\Delta P}_{\mathrm{insp}}\right)-{{\Delta P}_{\mathrm{insp}}}^{2}\cdot C\cdot \left[0.15\cdot x\right]\right\}$$

Once again utilizing the substitution ${x=t}_{\mathrm{slope}}/(R\cdot C)$ and making some minor rearrangements yields the final form of the linear model:

1 $${\mathrm{MP}}_{\mathrm{LM}}=0.098\cdot \mathrm{RR}\cdot \left\{{V}_{\mathrm{T}}\cdot \left({P}_{\mathrm{PEEP}}+{\Delta P}_{\mathrm{insp}}\right)-0.15\cdot {{\Delta P}_{\mathrm{insp}}}^{2}\cdot {t}_{\mathrm{slope}}/R\right\}$$

**Fig. 5:**
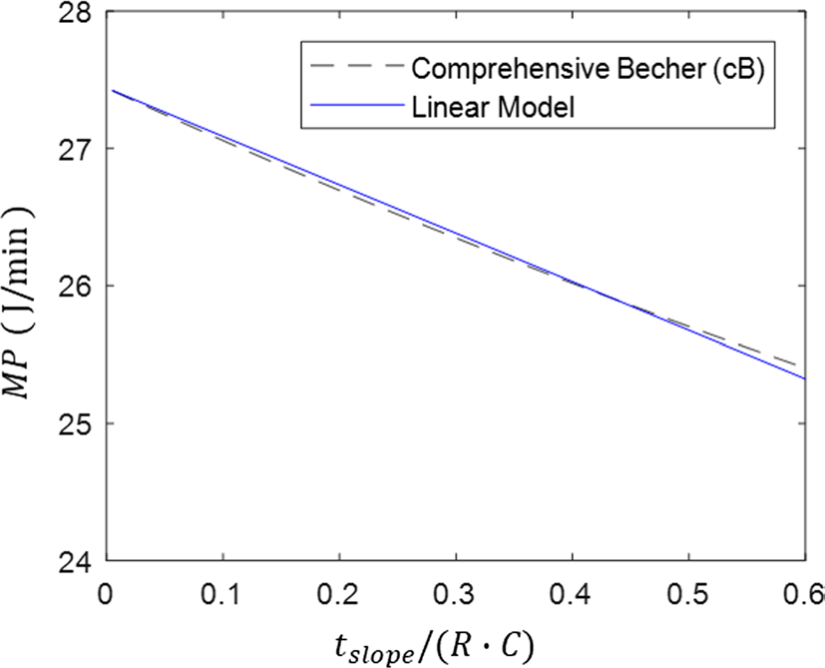
Comparison of mechanical power predicted by the comprehensive Becher, et al. equation and the linear model derived here. The two equations show excellent agreement over a realistic range of *t*_slope_/(*RC*) values

Figure 5 shows the mechanical power predicted by the original comprehensive Becher power equation compared to the power predicted by the linear model derived here. For this figure, ${V}_{\mathrm{T}}=0.5\,L$, ${\Delta P}_{\mathrm{insp}}=20\, {\mathrm{cmH}}_{2}\mathrm{O}$, ${P}_{\mathrm{PEEP}}=8\, {\mathrm{cmH}}_{2}\mathrm{O}$, $C=0.03\, \mathrm{L}/{\mathrm{cmH}}_{2}\mathrm{O}$, and $\mathrm{RR}=20\, \mathrm{breaths}/\mathrm{min}$.

### Data analysis

Percent error was calculated by first computing power for each patient data set using numerical integration [to obtain MP_ref_, Eq. (2)] and each of the four equations (Eqs. 1, 3–5). Percent error (*%*Error) is defined as the difference between the integrated value and the value estimated by a given equation, divided by the integrated value. For example, for a given patient data set (*i*) using the LM equation, the percent error would be

14 $$\%{\mathrm{Error}}_{\mathrm{LM},i}=({\mathrm{MP}}_{\mathrm{LM},i}-{\mathrm{MP}}_{\mathrm{ref},i})/{\mathrm{MP}}_{\mathrm{ref},i}$$

The discrete error values for all 50 patient data sets for a given equation were then used to calculate the average (μError) and standard deviation (StDevError) percent error values:

15 $$\mathrm{\mu Erro}{\mathrm{r}}_{\mathrm{LM}}=\frac{1}{N}\sum_{i=1}^{N}({\mathrm{MP}}_{\mathrm{ref},i}-{\mathrm{MP}}_{\mathrm{LM},i})/{\mathrm{MP}}_{\mathrm{ref},i}=\frac{1}{N}\sum_{i=1}^{N}\%{\mathrm{Error}}_{\mathrm{LM},i}$$

16 $${\mathrm{StDevError}}_{\mathrm{LM}}=\sqrt{\frac{1}{N}\sum_{i=1}^{N}{\left(\%{\mathrm{Error}}_{\mathrm{LM},i}-\mathrm{\mu Erro}{\mathrm{r}}_{\mathrm{LM}}\right)}^{2}}$$

Limits of Agreement (LoA) for the Bland–Altman plots in Fig. 1 were calculated for 95% agreement (± 1.96 standard deviation). The equations for the mean difference (μDif) and LoA between the integrated value for mechanical power and the equation-predicted value (for example, using the LM equation) are as follows:

17 $${\mathrm{\mu Dif}}_{\mathrm{LM}}=\frac{1}{N}\sum_{i=1}^{N}({\mathrm{MP}}_{\mathrm{LM},i}-{\mathrm{MP}}_{\mathrm{ref},i})$$

18 $${\mathrm{LoA}}_{\mathrm{LM}} =\pm 1.96\sqrt{\frac{1}{N}\sum_{i=1}^{N}{\left[({\mathrm{MP}}_{\mathrm{LM},i}-{\mathrm{MP}}_{\mathrm{ref},i})-\mathrm{\mu Di}{\mathrm{f}}_{\mathrm{LM}}\right]}^{2}}$$

Statistical analysis (paired samples two-tailed Student’s *t* test) and other calculations were performed using Matlab software (R2017a).

### Patient and ventilator parameters

Data used in this study were collected for a database to be used in future studies. Data were collected for any patient admitted to the medical ICU who needed mechanical ventilatory support between October 2020 and March 2021. This population included patients on different types of ventilatory support (e.g., PCV, VCV), so only data from patients on ventilators in PCV mode with nonzero rise time were used in this study. No patients who met these criteria were excluded. All data were collected in accordance to local IRB guidelines and study was approved by university institutional review board. All patients were on the same type of ventilators (Maquet Servo-i ventilators, Gothenberg, Sweden) on PCV. A 20 s capture of pressure, flow and tidal volume digital data (measured at 100 Hz) was downloaded to a PC card from the ventilator and used for analysis. Details on the patients and ventilator parameters can be found in Tables 1 and 2.

## Data Availability

Information on data sets can be obtained by contacting the corresponding author.

## Abbreviations

ARDS: Acute respiratory distress syndrome
BMI: Body mass index
*C*: Mechanical compliance
cB: Comprehensive Becher
*C*_dyn_: Compliance (dynamic)
COPD: Chronic obstructive pulmonary disease
IBW: Ideal body weight
LM: Linear model
LoA: Limits of agreement
MP_cB_: Mechanical power—calculated using the comprehensive Becher equation
MP_LM_: Mechanical power—calculated using our proposed linear model equation
MP_ref_: Mechanical power—calculated by integration of *P*–*V* loop
MP_sB_: Mechanical power—calculated using the simplified Becher equation
MP_vdM_: Mechanical power—calculated using the van der Meijden equation
*P*_aw_: Airway pressure
*P*_PEEP_, PEEP: Positive end expiratory pressure
PCV: Pressure-controlled ventilation
*R*: Flow resistance
*R*^2^: Coefficient of determination
RR: Respiratory rate
sB: Simplified Becher
StDevError: Standard deviation of percent error over all patient data sets
*t*_slope_: Inspiratory rise time
VCV: Volume-controlled ventilation
vdM: Van der Meijden
*V*_min_: Minute ventilation
*V*_T_: Tidal volume
Δ*P*_insp_: Pressure change from end expiration to end inspiration
μDif: Mean difference
μError: Mean percent error over all patient data sets
%Error: Percent error for a single patient data set
